# Supplementary material for: Unidirectional spin density wave state in metallic (Sr1−xLax)2IrO4
Source: Nat Commun. 2018 Jan 9;9:103. doi: 10.1038/s41467-017-02647-1 (PMC5760634; doi:10.1038/s41467-017-02647-1)
Supplement: Supplementary file 1 — Supplementary Information [file 41467_2017_2647_MOESM1_ESM.pdf]

Supplementary Information:

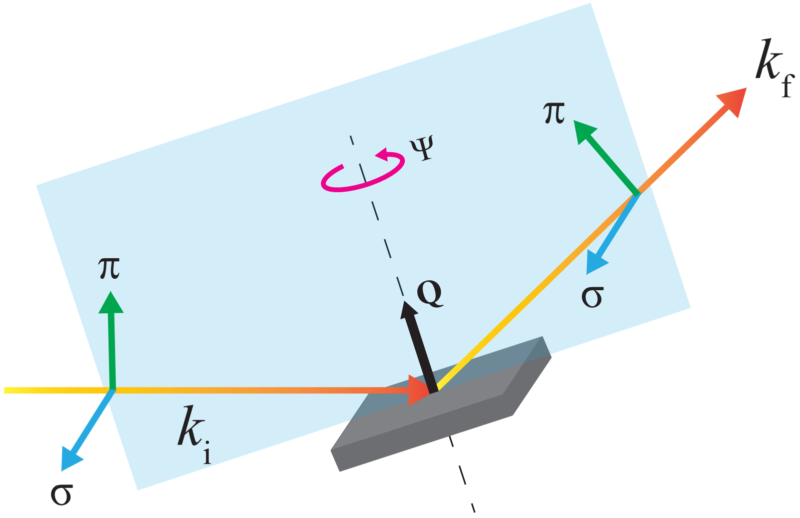

Supplementary Figure 1. Resonant elastic X-ray scattering (REXS) geometry. A vertical scattering plane of $(H, 0, L)$ or $(0, K, L)$ was employed. The incoming beam was horizontally polarized perpendicular to the scattering plane. Unless otherwise specified, all of the scattering data were collected in the polarization rotated $\sigma - \pi$ channel at temperature 10 K.

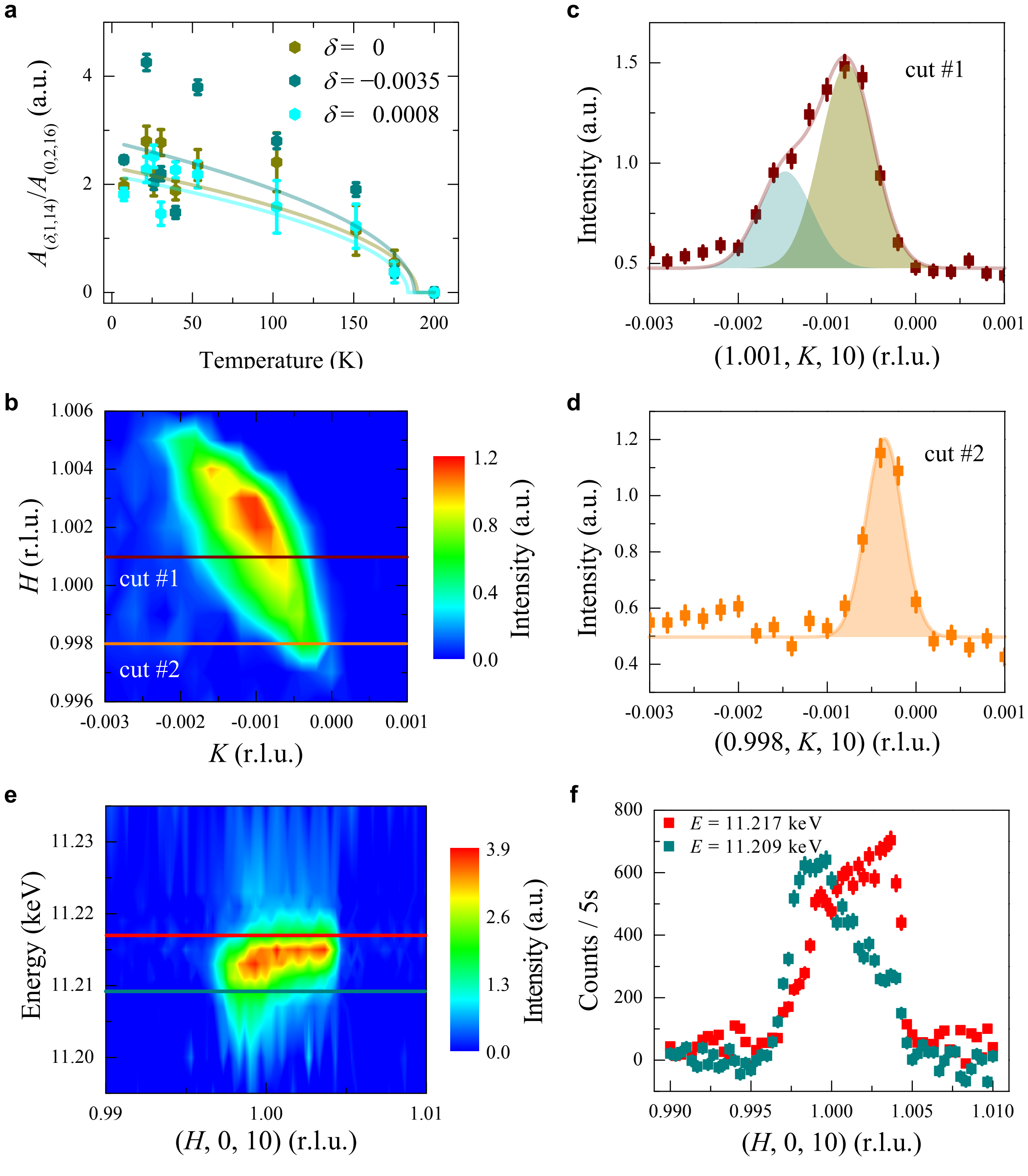

Supplementary Figure 2. Additional data for the $(\mathrm{Sr}_{1-x}\mathrm{La}_x)_2\mathrm{IrO}_4$ $x = 0.04$ sample. (**a**) Temperature dependence of the three peaks resolved in the H scan of $(0, 1, 14)$ of Fig. 3**c**. Panels (**b-f**) show data collected at $\mathbf{Q} = (1, 0, 10)$ or $(1, 0, 14)$ positions as Fig. 3**c**; however after the sample was rotated by 90°. (**b**) The detailed H, K map around the resulting $(1, 0, 10)$ reflection. Data from K scans through this map (cuts #1 and #2) are shown in panels (**c**) and (**d**). (**e**) Energy dependence of H scans about the $(1, 0, 14)$. Select H scans around $(1, 0, 14)$ at energies $E = 11.209$ keV (dark cyan) and $E = 11.217$ keV (red) are illustrated as lines with the corresponding data plotted in (**f**). Solid lines in (**a**), (**c**) and (**d**) are fits to the data as described in the text. Vertical error bars in (**a**) represent the errors of the ratio of the (δ, 1, 14) peak intensity to that of (0, 2, 16) peak, which are both accompanied by statistical errors of 1 standard deviation (s.d.). All vertical error bars in (**c-d**, **f**) represent 1 s.d. statistical errors.

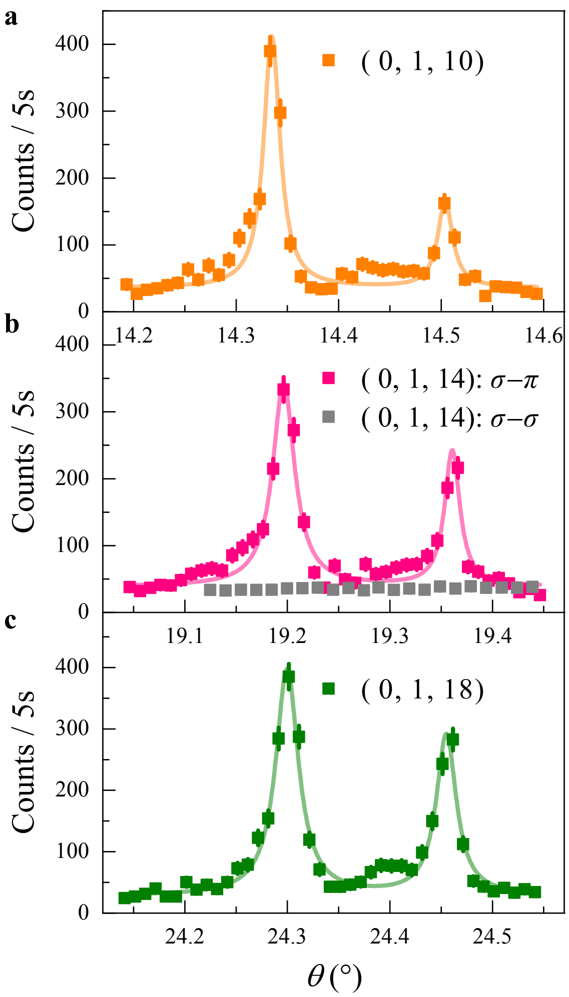

Supplementary Figure 3. Additional incommensurate peaks about the $(0, 1, 4N + 2)$ (N= 2, 3, 4) magnetic positions are shown as θ scans in the $x = 0.041$ sample. Panel **b** also includes the $\sigma - \pi$ and $\sigma - \sigma$ comparison of the incommensurate scattering about the $(0, 1, 14)$ magnetic peak. Solid lines are Lorentzian fits to the peaks. Vertical error bars represent 1 s.d. statistical errors.

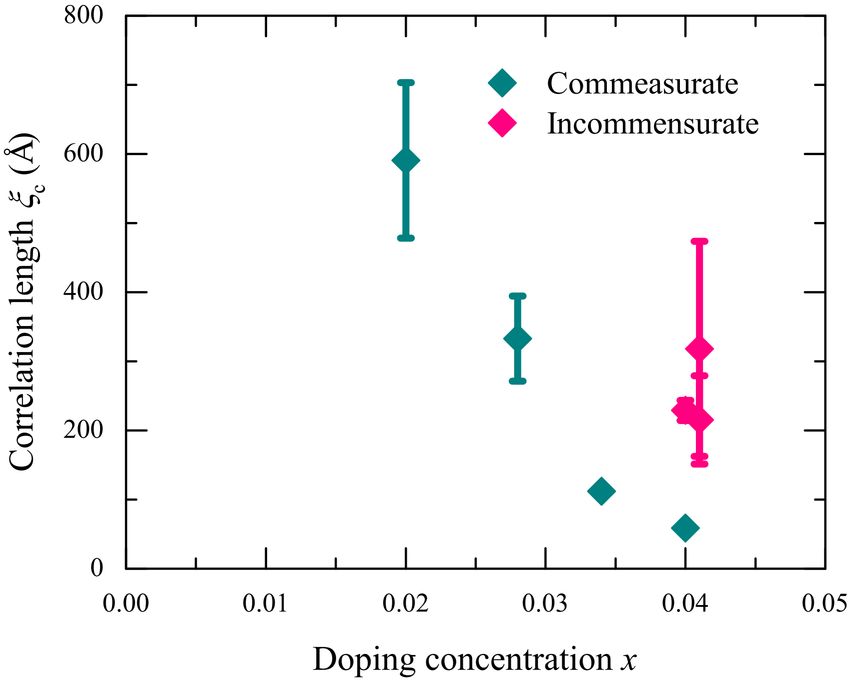

Supplementary Figure 4. Resolution deconvolved c-axis correlation lengths for the saturated antiferromagnetic states ($T = 10$ K) are plotted as a function of doping x in $(Sr_{1-x}La_x)_2IrO_4$. Below $x \approx 0.02$, the magnetic peaks are resolution limited, indicating the true long range nature. With increasing doping, the correlation length decreases drastically before the emergence of the incommensurate order above the critical doping $x = 0.04$. Vertical error bars originate from the uncertainties in deconvolving the magnetic peak full width at half maximum (FWHM) $\mathit{\Gamma}_{\mathrm{Mag}}$ from the experimental resolution peak FWHM $\mathit{\Gamma}_{\mathrm{Res}}$ and are obtained through error propagation by taking the Fourier transforms of the deconvolved Lorentzians in describing the magnetic peaks.

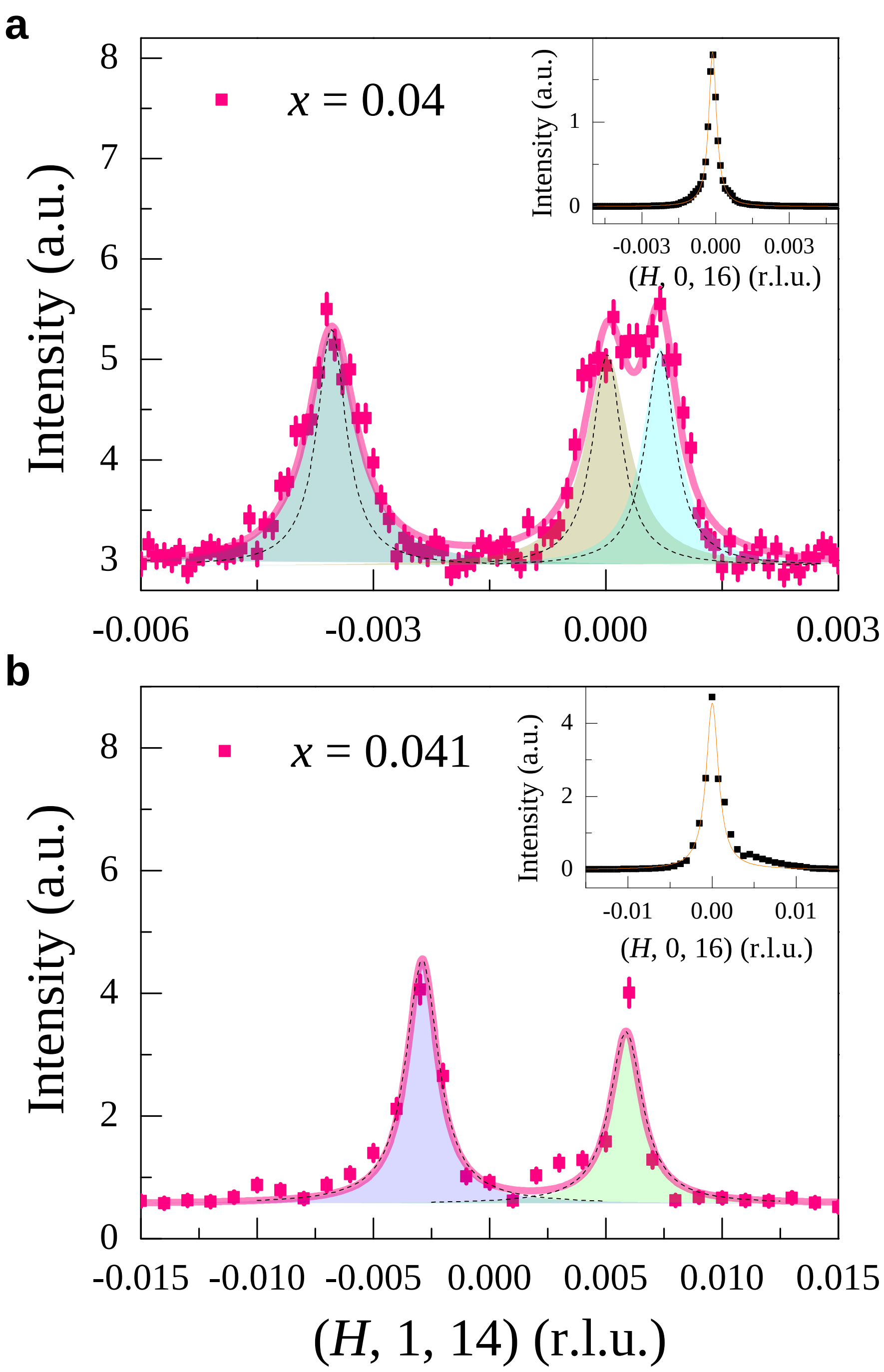

Supplementary Figure 5. Comparison of magnetic and charge peak widths for the $x = 0.04$ (**a**) and $x = 0.041$ (**b**) samples. Upper right insets in (**a-b**) show the charge peak widths and these widths are over-plotted as dashed lines inside of the magnetic peaks for reference. The magnetic peaks around (0, 1, 14) are reproduced from Fig 3**c** and Fig 4**c**. Vertical error bars represent 1 s.d. statistical errors.

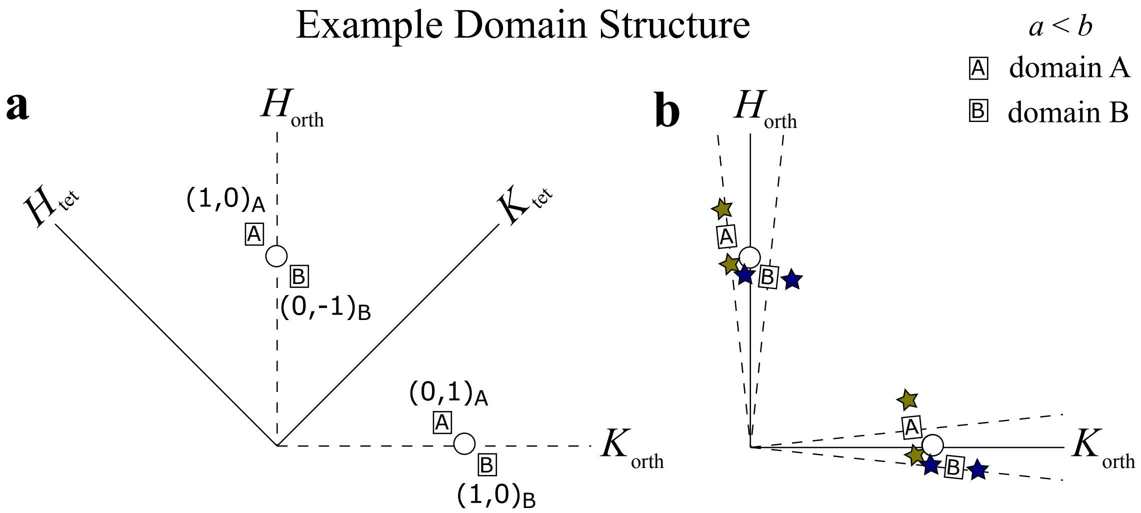

Supplementary Figure 6. Illustration of how domain structure can alter apparent incommensurate positions. (**a**) Tetragonal and orthorhombic axes for the IrO_2 planes. Tetragonal here denotes the smaller unit cell where H and K point along the Ir-Ir bond direction while orthorhombic denotes the larger unit cell with H and K parallel to the bond diagonals. Two potential domains A and B are marked if the lattice were to be subtly orthorhombic. (**b**) H and K axes again in the larger cell are depicted with circles denoting the aligned (1,0) and (0,1) positions assuming $a_{\mathrm{orth}} = b_{\mathrm{orth}}$ and the relative orientations of the A and B domains are also illustrated under this assumption. Stars denote the unidirectional splitting from each domain with spin modulation along its local H_{orth}-axis.

$(Sr_{1-x}La_x)_2IrO_4$	Instrument	L_3 (keV)	Mosaic (°)
$x = 0.02$	CHESS,A2	11.217	0.09(1)
$x = 0.028$	CHESS,C1	11.215	0.05(1)
$x = 0.04$	APS,6-ID-B,C	11.214	0.008(1)
$x = 0.041$	CHESS,A2	11.217	0.024(1)

Supplementary Table I. Summary of samples measured, their crystal mosaic, and corresponding beam lines. The relative shifts in energy calibration for each beam line are also tabulated by denoting the observed Ir L_3 absorption edge energies.

$(Sr_{1-x}La_x)_2IrO_4$	$x = 0$	$x = 0.028$	$x = 0.04$
Lattice a (Å)	5.4928(5)	5.5037(11)	5.504(6)
Lattice c (Å)	25.784(2)	25.807(6)	25.77(3)
R factor	0.0690	0.0300	0.0772
GoF	0.998	1.109	1.09
Atom U_{iso} (Å^2)	$x = 0$	$x = 0.028$	$x = 0.04$
Ir (8a)	0.003	0.001	0.002
Sr/La (16d)	0.007	0.006	0.007
O1 (16d)	0.010	0.008	0.008
O2 (16f)	0.009	0.009	0.010

Supplementary Table II. Summary of single crystal refinement data of select samples in $(Sr_{1-x}La_x)_2IrO_4$. The space group $I4_1/acd$ was adopted for refinement.

Supplementary Note 1: Scattering geometry

Supplementary Figure 1 shows a schematic depicting the scattering geometry employed in the experiment, using conventional notations for the photon polarization vectors [1]. A vertical scattering plane of $(H, 0, L)$ or $(0, K, L)$ was employed, and the incoming beam was horizontally σ polarized. Magnetic scattering was isolated in the polarization rotated $\sigma - \pi$ channel, and unless otherwise specified, data were collected in $\sigma - \pi$ scattering channel at temperature 10 K.

Once the vertical scattering plane $(H, 0, L)$ was defined, only the $(0, 1, 4N + 2)$ or $(1, 0, 4N)$ (N = integer) type magnetic reflections are resolvable[2–4]. $(1, 0, 4N + 2)$ and $(0, 1, 4N)$ type magnetic reflections arising from the other magnetic domain (with rotated moments and modified interplane phasing) are hidden due to the azimuthal dependence of the scattering intensity. Correspondingly, when the sample is rotated by 90° with respect to the crystal's c-axis (Fig. 3**f** and Supplementary Figures 2(**b-f**)), then the $(0, K, L)$ scattering plane is accessed and only those previously silent $(1, 0, 4N + 2)$ or $(0, 1, 4N)$ type magnetic peaks from the other domain set will contribute. In short, only one domain of the crystal will contribute to scattering either in the $(H, 0, L)$ or $(0, K, L)$ vertical scattering planes as a result of the azimuthal dependence of the magnetic intensity. Finally, the incident X-ray beam has a large horizontal divergence perpendicular to the scattering plane. As a result, the instrumental resolution is broadened along this direction (e.g. broad along K for the $(H,\ 0,\ L)$ scattering plane), as shown in Figs. 3(**a-b**) and Figs. 4(**a-b**).

Supplementary Note 2: Single crystal refinement

As a further consistency check, single crystal X-ray diffraction measurements and structural refinements were performed on select samples used in synchrotron experiments. Crystals were mounted on a glass fiber and transferred to a Bruker Kappa APEX II diffractometer with a Mo K_α source. The APEX2[5] program was used to determine the unit cell parameters and data collection was performed using 10 sec / frame and 0.5 deg./ frame Omega scan. Data were collected at room temperature and refined using the SHELXTL[6] program. The similar R factors, Goodness of Fit (GoF) and comparable atomic displacement parameters (ADP) indicate consistent crystal qualities of doped samples when compared to the parent system.

Supplementary Note 3: Additional data collected at 6-ID-B, APS

Additional data collected on the $x = 0.04$ sample at the 6-ID-B beam line are shown in Supplementary Figure 2. The temperature dependence of the three peaks identified about the $(0, 1, 14)$ magnetic zone center from Figure 3**c** are plotted in Supplementary Figure 2**a** for comparison. Both the commensurate and incommensurate magnetic peaks disappear at the same temperature, which is estimated to be $T_{\text{AF}} = 188 \pm 15$K.

Supplementary Figures 2(**b-f**) show data obtained after the sample was rotated counter-clockwise by 90° with respect to the crystal c-axis. Following this rotation, the scattering plane becomes (0,K,L), and Supplementary Figure 2**b** shows the H, K map of scattering intensities collected about the $(1, 0, 10)$ magnetic zone center at the temperature 10 K. It should be noted that for the parent magnetic structure, the $(1, 0, 10)$ peak intensity is interpreted as arising from the second allowed magnetic domain in the crystal. After the sample rotation, the resolution along K (estimated to be 0.0005 $r.l.u.$) becomes much better than that along H (estimated to be 0.0023 $r.l.u.$) rendering any small incommensurate splitting along H difficult to resolve. The K scan in Fig. 3**f** shows only one peak, demonstrating no equivalent splitting along K.

A remaining question concerns the degree to which the unidirectional incommensurate splitting along H is resolvable in the rotated $(1, 0, 10)$ zone. Cuts taken through the resolution broadened peak (indicated by lines marked #1 and #2 in Supplementary Figure 2**b**) are plotted in Supplementary Figures 2(**c-d**). These cuts suggest the presence of two weakly resolved components split along H. This implies that the incommensurate splitting is still present along H after the sample's rotation, yet it is largely blurred by the instrumental resolution.

The observation of weakly resolvable incommensurate splitting in the 90° rotated zone is further supported by H scans collected through the equivalent $(1, 0, 14)$ magnetic zone at a range of different energies plotted in Supplementary Figures 2(**e-f**). As discussed in the main manuscript text, the resonance energies of the commensurate and incommensurate magnetic peaks of this sample are shifted from one another by approximately 1.5 eV. By tuning to each resonance energy individually and performing H scans (denoted by lines in Supplementary Figure 2**e**), subtle

shifts between convolved components forming the single peak can potentially be resolved. Supplementary Figure 2**f** shows the resulting scans at energy $E = 11.217$ keV, where the right shoulder of the broad peak is promoted, and at energy $E = 11.209$ keV where the left shoulder of the peak is enhanced. This subtle offset in the spectral weight of the peak with changing energy suggests the presence of the lower energy incommensurate peaks convolved within the resolution broadened peak.

A rough estimation of the splitting between the convolved peaks in this rotated zone shows the separation between two components along H to be about 0.005 *r.l.u.*, close to the measured splitting of 0.0045 *r.l.u.* in the $(0, K, L)$ zone. This combined analysis strongly suggests that the incommensurate peaks split along H are still present in the scattering data after the sample is rotated by 90°. This is consistent with the incommensurate state being unidirectional and occurring only along H direction, regardless of the magnetic zones chosen or the relative orientation of the sample.

Supplementary Note 4: Additional data collected at A2, CHESS

Additional θ scans through the $(0, 1, 4N + 2)$ (N= 2, 3, 4.) magnetic peaks of the $x = 0.041$ sample are shown in Supplementary Figure 3. Here, θ scans are nearly parallel to the H axis. The two incommensurate peaks are resolved in all of the zones examined, and the splitting along only H remains unidirectional. Supplementary Figure 3**b** also includes the $\sigma - \pi$ and $\sigma - \sigma$ comparison of the $(0, 1, 14)$ magnetic peak. The incommensurate scattering signal appears only in the $\sigma - \pi$ channel.

Supplementary Note 5: Comparison of spin-spin correlation lengths

The out-of-plane correlation lengths along c-axis were determined at 10 K for all the samples studied and are plotted in Supplementary Figure 4. For La concentrations below $x \approx 0.02$, the magnetic peaks are resolution-limited in all directions, indicating true long-range order[7]. This agrees with the previously reported long-range order in neutron diffraction studies exploring concentrations up to $x = 0.02$[7]. Upon increasing electron doping, the correlation length along c decreases drastically before the emergence of the incommensurate order beyond the critical doping $x = 0.04$. The decrease in correlation length along c with increasing doping suggests the transition from true three-dimensional long-range order toward a quasi two-dimensional state.

Upon electron doping up to $x = 0.041$, the in-plane correlation lengths remain long range, as suggested by the comparable magnetic (shaded peaks) and resolution peak (dashed lines) widths in Supplementary Figure 5. At this limit comparing the crystallinity and resolution-convolved widths of the charge peaks (at different $\mathbf{Q}$s) with the slightly broader magnetic peaks may give somewhat misleading correlation lengths, and instead the minimum spin-spin in-plane correlation length is quoted in the main text. Thus close to the doping threshold of $x = 0.04$, the antiferromagnetic correlations are reflective of quasi two-dimensional short-range order. The gradual transition between these extremes is summarized in the phase diagram presented in the main text (Fig. 1).

Supplementary Note 6: Illustration of possible domain structure

As mentioned in the main text, the most likely origin of the apparent asymmetry of the incommensurate peaks about the magnetic zone center is a subtle underlying orthorhombic symmetry of the lattice. As the experimentally observed spin modulation is unidirectional and breaks the four-fold rotational symmetry of the lattice, the underlying crystal structure is necessarily orthorhombic or lower in symmetry. If this orthorhombicity is small, it is often difficult to resolve due to lattice twinning effects[8], yet alignment of the crystal using a apparent tetragonal lattice can nevertheless yield offsets in the observed incommensurate peaks.

As an illustration, a number of twinning scenarios are possible with typical in-plane orthorhombic twinning patterns of (a_{short}, b_{long},), (-a_{short}, b_{long}), (b_{long}, a_{short}), (-b_{long}, a_{short}). We illustrate how this can generate an apparent offset in magnetic scattering by considering a simple two-domain structure possible in an orthorhombic lattice shown in Supplementary Figure 6. Alignment based on the charge peaks assuming a tetragonal lattice will roughly choose an average position between the two domains (open circles) and generate an apparent offset for each domain's magnetic zone center. For the small offset we see in our measurements of the incommensurate scattering in $(Sr_{1-x}La_x)_2IrO_4$

(0.0015 *r.l.u.*), this would imply a rotation of domains by only 0.085° and an underlying orthorhombicity of $(a-b)/(a+b) = 0.001499$. In this simple picture, measurements may only resolve either domain A or domain B due to a combination of the magnetic structure factor and the relative orientation/texture of spins in each domain. For instance, only the (1 ,0) or (0, 1) zone centers are allowed for $L = 4N$ or $4N + 2$ planes respectively, and further selection rules based on the currently unresolved magnetic domain structure and the spin modulation/orientation within a given domain (e.g. sinusoidal versus cycloidal order) will also arise. Together these factors constitute the likely source of the asymmetric splitting observed in the small incommensurate ordering wave vectors of the $x = 0.04$ and $x = 0.041$ crystals.

Supplementary References

[1] Boseggia, S. *et al.* Locking of Iridium magnetic moments to the correlated rotation of Oxygen octahedra in Sr_2IrO_4 revealed by X-ray resonant scattering. *J. Phys. Condens. Matter* **25**, 422202 (2013).
[2] Kim, B. J. *et al.* Phase-sensitive observation of a spin-orbital Mott state in Sr_2IrO_4. *Science* **323**, 1329–1332 (2009).
[3] Dhital, C. *et al.* Neutron scattering study of correlated phase behavior in Sr_2IrO_4. *Phys. Rev. B* **87**, 144405 (2013).
[4] Ye, F. *et al.* Magnetic and crystal structures of Sr_2IrO_4: A neutron diffraction study. *Phys. Rev. B* **87**, 140406 (2013).
[5] APEX2 V2014.11-0 Software Users Guide, Bruker Analytical X-ray Systems, Inc., Madison, WI 2014.
[6] Sheldrick, G. M. SHELXTL, Version 6.12, Bruker Analytical X-ray Systerms, Inc.; Madison, WI 2001.
[7] Chen, X. *et al.* Influence of electron doping on the ground state of $(Sr_{1-x}La_x)_2IrO_4$. *Phys. Rev. B* **92**, 075125 (2015).
[8] Hogan, T. *et al.* Structural investigation of the bilayer iridate $Sr_3Ir_2O_7$. *Phys. Rev. B* **93**, 134110 (2016).
